# Association between Sex Hormone and Blood Uric Acid in Male Patients with Type 2 Diabetes

**DOI:** 10.1155/2017/4375253

**Published:** 2017-10-03

**Authors:** Wen Cao, Ren-Dong Zheng, Shu-Hang Xu, Yao-Fu Fan, Hong-Ping Sun, Chao Liu

**Affiliations:** ^1^The First Clinical Medical College, Nanjing University of Chinese Medicine, Nanjing 210013, China; ^2^Endocrine and Diabetes Center, Affiliated Hospital of Integrated Traditional Chinese and Western Medicine, Nanjing University of Chinese Medicine, Nanjing 210028, China

## Abstract

The association between serum uric acid (SUA) level and sexual dysfunction in patients with diabetes is not well characterized. Type 2 diabetes mellitus (T2DM) causes metabolic disorders, including abnormal serum uric acid (SUA) levels. In this study, we enrolled 205 male patients with T2DM and investigated the relationship between sex hormone levels and SUA. Patients were divided into four groups based on SUA quartiles. On the other hand, based on the total testosterone (TT) level, patients were divided into three groups; SUA and other laboratory indices were determined. Increase in SUA level was significantly associated with decreased levels of TT, luteinizing hormone, follicle-stimulating hormone, sex hormone-binding globulin, and increased levels of dehydroepiandrosterone, age, body mass index (BMI), waist circumference, glycated hemoglobin, serum creatinine, and HOMA-IR levels. SUA, waist circumference, BMI, and HOMA-IR showed a negative correlation with TT level, while age showed a positive correlation with TT level. SUA and body mass index were found to be risk factors for gonadal dysfunction. Therefore, we conclude that hypogonadism of male patients with T2DM is related to SUA level.

## 1. Introduction

Hypogonadism is characterized by decreased sexual frequency, sexual ability, and morning libido. The diagnosis of hypogonadism is based on symptom score and serum sex hormone levels. Total testosterone level < 12 nmol/L is suggestive of hypogonadism; patients with total testosterone levels ≤ 8 nmol/L typically benefit from testosterone treatment [1, 2]. Studies have demonstrated that hypogonadism may occur in men with metabolic diseases [3, 4]. Low levels of serum testosterone (T) have been documented in male patients with metabolic syndrome (MS). The underlying mechanism may be dietary-induced hypothalamic inflammation, which reduces the release of gonadotropin-releasing hormone (GnRH) and correlates with age and body mass index (BMI) [5, 6].

In recent years, the incidence of hyperuricemia has gradually increased. In addition to the injury to the joints and kidney, hyperuricemia leads to a variety of metabolic diseases [7, 8]. Patients with type 2 diabetes often have other coexisting metabolic disorders, particularly hyperuricemia. Our previous study found that obese males with type 2 diabetes usually develop hypogonadism. Al et al. [9] also reported a close association of hypogonadism with insulin resistance [10] and lipid metabolism [11], which is consistent with our results. Hyperuricemia-related diseases, such as obesity, diabetes, and hyperlipidemia, increase the risk of gonadal dysfunction. Studies have shown that testosterone supplementation can improve body composition and glycolipid metabolism in younger subjects and in those with metabolic disturbances [12]. Testosterone supplementation was shown to improve body weight and waist circumference in patients with type 2 diabetes [13]. In this study, we sought to identify risk factors for hypogonadism by analyzing the relationship between sex hormone levels and blood uric acid levels in men with type 2 diabetes. Our findings may provide evidence for the prevention and treatment of hypogonadism.

## 2. Subjects and Methods

### 2.1. Subjects

A total of 1026 male patients with type 2 diabetes who were hospitalized at the Jiangsu Province Hospital of TCM and Western Medicine, Nanjing University of Chinese Medicine, between January 2013 and June 2016 were eligible for inclusion.

**Figure 1:**
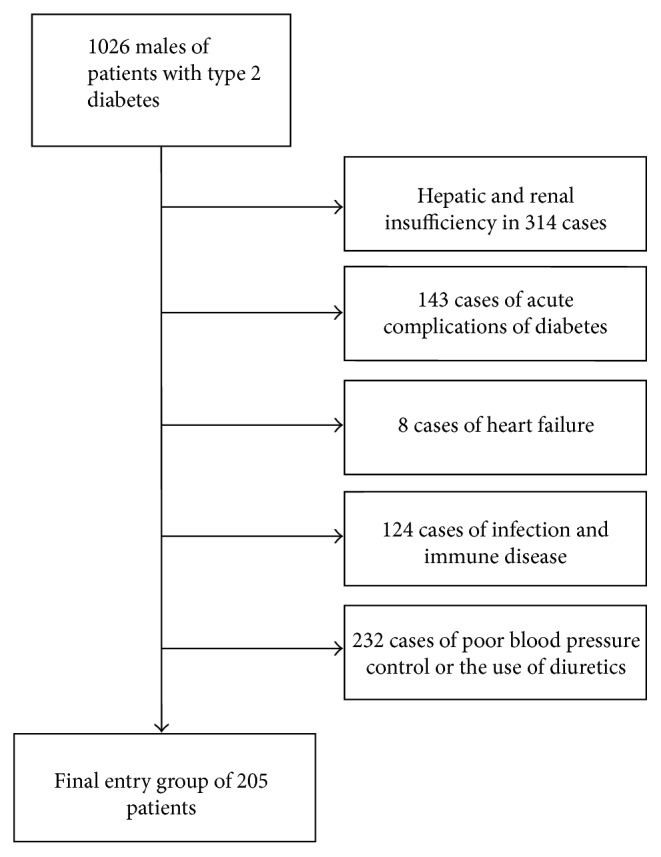
Flowchart showing criteria for patient selection.

The following are the exclusion criteria: (1) acute and chronic kidney disease; (2) cardiac insufficiency; (3) acute diabetic complications; (4) history of sex gland diseases; (5) infectious and autoimmune diseases; (6) history of hypertension and use of diuretics, or patients with uncontrollable blood pressure (≥140/90 mmHg). Based on the exclusion criteria, 314 patients with liver or renal insufficiency, 143 patients with acute diabetic complications, 8 patients with heart failure, 124 patients with infectious and immune diseases, 232 patients with uncontrolled high blood pressure, or those using diuretics were excluded. Finally, 205 patients were included in the study (Figure 1). All patients were aged between 27 and 81 years and conformed to the diabetes diagnostic criteria set by the World Health Organization (WHO) in 1999. Depending on the serum uric acid levels tested on the 3 points (260, 308, and 385 *μ*mol/L) of tangency quarterback method (Q1 = 0–25%, Q2 = 26–50%, Q3 = 51–75%, and Q4 = 76–100%), patients were divided into four groups: group Q1 (133–260 *μ*mol/L); group Q2 (260–308 *μ*mol/L); group Q3 (308–380 *μ*mol/L); and group Q4 (380–602 *μ*mol/L). Symptom scores and sex hormone levels were used to evaluate gonad function. Written informed consent was obtained from all subjects, and the research plan was approved by the ethics committee.

### 2.2. Methods

#### 2.2.1. General Indices

Body weight, blood pressure, and BMI were measured. All patients were made to stand with feet separated as wide as the distance between the two shoulders. The waist circumference was measured in the horizontal plane midway between the lowest rib and the iliac crest [14]. History of smoking and hypertension was noted.

#### 2.2.2. Blood Indices

Blood samples were obtained between 6 and 7 am after overnight fasting for 8–10 hours. Indices included fasting blood glucose (FBG); glycated hemoglobin (HbA_1c_); blood urea nitrogen (BUN); creatinine (Cr); serum uric acid (SUA); sex hormones [total testosterone (TT), luteinizing hormone (LH), follicle-stimulating hormone(FSH), estradiol, sex hormone-binding globulin (SHBG), and dehydroepiandrosterone (DHEA)]; and fasting insulin (FINS). Then, 200 mL of water containing 75 g of anhydrous glucose was administered. Two hours later, blood samples were collected to determine postprandial blood glucose. The hormone indices were analyzed by a chemiluminescence method; blood glucose level was determined by a glucose oxidase method; HbA_1c_ level was determined by a chromatography method; and uric acid level was determined by an enzymatic method. To measure serum SHBG, total testosterone, and serum albumin levels, a software was used to calculate the bioactive testosterone (BT) and free testosterone (FT) levels (http://www.issam.ch/). The following is the homeostasis model assessment-insulin resistance index (HOMA-IR) calculation formula: fasting blood glucose level(mmol/L) × fasting insulin level (mIU/L)/22.5.

### 2.3. Statistical Processing

SPSS16.0 software was used for the statistical analysis. Normally distributed variables are expressed as mean ± standard deviation, and between-group differences assessed by a single-factor analysis of variance (ANOVA). Nonnormally distributed variables are expressed as median, and between-group differences assessed by the Kruskal-Wallis test. Correlations were assessed using Spearman's method for nonnormally distributed data, and Pearson's method was used for normally distributed data. Multiple regression analysis was performed to identify correlates of TT level. *p* < 0.05 was considered statistically significant.

## 3. Results

### 3.1. Clinical Characteristics of the Participants

Significant differences were observed between the four groups with respect to age, BMI, waist circumference, HbA_1c_, creatinine, and HOMA-IR (*p* < 0.05 for all). No significant between-group differences were observed with respect to blood pressure, fasting blood glucose level, and BUN (*p* > 0.05) (Table 1).

### 3.2. Comparison of Sex Hormone Levels between Four Groups

**Figure 2:**
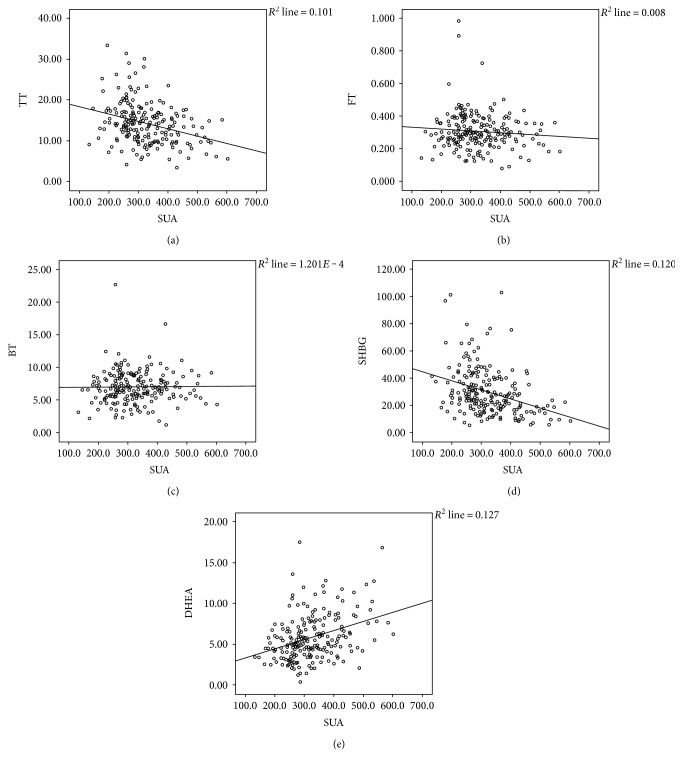
Hormone scatter diagram. Scatter plot showing correlation of SUA levels with TT, FT, BT, SHBG, and DHEA. With the rise of SUA, TT, and SHBG decreasing significantly, DHEA significantly increased. SUA levels showed a negative correlation with TT and SHBG levels (*r* = −0.308, *p* < 0.001 and *r* = −0.379, and *p* < 0.001, resp.) and a positive correlation with DHEA (*r* = 0.352, *p* < 0.001), FT (*r* = −0.090, *p* = 0.199), and BT (*r* = 0.011, *p* = 0.876) levels. TT: total testosterone; BT: bioactive testosterone; FT: free testosterone; E2: estradiol; SHBG: sex hormone-binding globulin; DHEA: dehydroepiandrosterone; SUA: serum uric acid.

Increased levels of SUA were significantly associated with a decrease in TT, LH, FSH, and SHBG levels (*p* < 0.05) and increase in DHEA levels (*p* < 0.05). No significant between-group difference was observed with respect to levels of bioactive testosterone (BT), free testosterone (FT), and estradiol (E2) (*p* > 0.05) (Table 2).  Figure 2 shows the scatter plot of TT, FT, BT, SHBG, and DHEA levels.

### 3.3. Comparison between Different TT Groups

Owing to the observed inverse association between TT and SUA levels, we categorized patients into three groups based on TT levels (<8 nmol/L; between 8 nmol/L and 12 nmol/L; and >12 nmol/L) to further characterize the association. The results showed that SUA, BMI, waist circumference, and HOMA-IR were significantly different between the three groups (*p* < 0.05) (Table 3).

### 3.4. Multivariate Regression Analysis of Male Hypogonadism

Multivariate regression analysis was performed to assess the correlation between TT levels and related indicators. SUA, waist circumference, BMI, and HOMA-IR showed a negative correlation with TT levels, while age showed a positive correlation with TT levels (*p* < 0.05) (Table 4). In the multivariate regression model, total testosterone level was included as the dependent variable, while age, waist circumference, BMI, HOMA-IR, and SUA were included as independent variables. Multivariate regression analysis revealed that BMI and SUA were risk factors for gonadal dysfunction (Table 5).

## 4. Discussion

Uric acid is the metabolic end product of nucleic acid purines *in vivo* (including nucleic acids in food) that is mainly excreted through renal excretion. Increase in SUA levels is generally caused by increased uric acid production and/or reduced excretion. The level of mammalian serum uric acid ranges between 1 and 3 mg/dL [15]. In recent years, the incidence of hyperuricemia (HUA) has increased gradually. A cross-sectional study of residents in Brazil in 2012 showed that the prevalence of HUA is 13.2% [16]. The prevalence of HUA was 25.8% according to a survey covering 9914 residents in Japan [17]. Our survey shows that the prevalence of HUA in rural residents in North and Northeast China is 10.9% [18].

The present study investigated 205 male patients with diabetes, of which 70 (34.2%) patients had TT levels < 12 nmol/L. Among healthy male individuals, the reported percentage of individuals with sexual dysfunction varies between 20 and 80% [19–21]. In an Australian cross-sectional study of 1089 patients with type 2 diabetes, 36.5% patients had hypogonadism [10]. Type 2 diabetes is likely to trigger a late onset of hypogonadism.

We found a significant correlation between SUA and TT levels. As the SUA level increased, testosterone levels decreased and the SUA level of patients with low TT was significantly increased.

Accounting for about 95% of the total sex hormone, testosterone is a major androgen mainly synthesized by testicular interstitial cells, as well as the reticular zone of adrenal cortex. Low testosterone level causes a series of physical changes, such as obesity, muscle hypertrophy, lipid metabolism disorders, osteoporosis, poor immunity, cardiovascular illness, and nervous dysfunction.

Epidemiological studies have shown that the incidence of hyperuricemia in men is higher than that in women [22, 23]. In the present study, an inverse association was observed between TT levels and uric acid levels among men with type 2 diabetes. A previous study has found decreased testosterone and estradiol synthesis in male patients with gout; patients with gouty kidney disease and gouty arthritis showed a significant decrease in testosterone levels [24]. The reasons may include (1) crystallization of uric acid in the testicular tissue causing oxidative damage [25] and (2) insulin resistance that can be reduced by low testosterone levels [26], results in reduced secretion of uric acid in renal tubular epithelial cells after absorption and the renal excretion of uric acid [27]. Therefore, the decline in body testosterone levels can lead to elevated serum uric acid levels; (3) testosterone promotes synthesis of protein and nucleic acids; decreased testosterone levels reduce protein synthesis and increase the level of endogenous purine, which causes hyperuricemia. Further research is needed to reveal the correlation between SUA and testosterone.

For patients with gonadal dysfunction, low hormone levels will stimulate hypothalamus-pituitary gonadotropin secretion. In early-stage type 2 diabetes, testosterone levels can be compensated to maintain the normal level. In elderly individuals, patients with advanced stage of type 2 diabetes and vasculopathy, compensatory function gradually declines, which results in low levels of gonadal hormones. We observed that LH and FSH decreased with elevated uric acid levels and lowered gonadal hormone levels. As TT levels decrease, patients may develop sexual dysfunction. The reason may be that hyperuricemia can lower LH levels and reduce the synthesis of testosterone and estrogen [28].

This study found that SUA was negatively correlated with SHBG and positively correlated with DHEA. SHBG specifically binds to sex hormones, participates in its transport, and regulates the concentration of biologically active sex hormones in the blood [29, 30]. Hyperuricemia can reduce the level of insulin-like growth factors binding to protein 3 and reduce the level of SHBG [31]. Studies conducted overseas have shown that postmenopausal women with increased SUA levels experience decline in sex hormone levels [32]. However, similar studies conducted on men are rare. DHEA, an adrenal steroid hormone abundant in human blood [33], is a biomarker of the hypothalamic-pituitary-adrenal (HPA) axis activity [34–36]. It seems that sex hormones primarily influence the SUA concentration via renal UA excretion [37–39].

We found that HOMA-IR levels in patients with higher SUA levels were significantly higher than those in patients with lower SUA levels, which suggests a direct association between SUA levels and insulin resistance. Long-term hyperinsulinemia can interfere with carbohydrate metabolism and weaken GA3PDH (glyceraldehyde-3-phosphate dehydrogenase) activity, which promotes the glycolytic metabolism of intermediate synthesis of ribose-5-phosphate (R-5-P), phosphoric acid (PPRP), and uric acid [40]. Insulin resistance can increase the synthesis of fat in the liver, which leads to disordered purine metabolism and consequent increase in SUA level. Further, too much insulin action can reduce excretion of uric acid in the renal tubules, which in turn leads to hyperuricemia. Insulin resistance is strongly associated with hyperuricemia [41], and the two can influence each other. Cruz-Dominguez et al. [42] found an inverse correlation between SUA levels and insulin sensitivity. We found that insulin resistance is an independent risk factor for male sexual dysfunction, which may be mediated via the following mechanisms: (1) insulin stimulates the gonadotropin-releasing hormone expression in the nerves of the hypothalamus and its subsequent secretion [43]. Insulin resistance decreases GnRH secretion, which causes a decrease in the concentration of LH and TT; (2) decrease in hormone levels in male patients leads to a rapid increase in body weight which further aggravates lipid metabolism disorders. This is the root cause of metabolic syndrome in patients with increased insulin resistance; (3) testosterone secretion can reduce the circulation of nonaromatic fatty acids and improve insulin sensitivity. Testosterone level decrease is also related to insulin sensitivity.

We found that obesity reduces sexual function as the SUA level significantly increases. The higher the SUA level, the higher the BMI and waist circumference, which suggests that uric acid and obesity are closely related. Patients with hyperuricemia tend to have higher BMI. This is associated with the acceleration of purine synthesis and increased uric acid production.

The higher the level of uric acid, the higher the Cr, which suggests that uric acid is a predictor of kidney disease. Uric acid induces endothelial cell damage by increasing intracellular oxidative stress and by upregulating C-reactive protein expression and intracellular NO activity. In the kidney, uric acid is known to induce renal renin expression, enhance the activation of RAAS system, and initiate renal afferent arteriolar sclerosis, glomerular hypertrophy, and atherosclerosis [44, 45]. Renal dysfunction was shown to cause hypothalamic pituitary gonadal axis dysfunction and to decrease testosterone secretion [46]. Decreased testosterone levels can weaken normal sexual initiation and sexual activity [47]. High SUA levels in patients with type 2 diabetes may be highly suggestive of gonad hypofunction.

However, this study has some limitations. First, this is a single-center study; our findings may not reflect the situation in other regions. In the future, we hope to expand the research area and observe more people. Second, we judged hypogonadism solely based on the testosterone levels and did not include clinical evaluation of patients; it is likely that some patients have clinical symptoms of sexual dysfunction despite no significant decrease in testosterone levels. These patients should be studied. Third, the small sample size limits the statistical power of the analysis. Further studies with a large number of participants are required to draw more definitive conclusions.

## 5. Conclusion

Hypogonadism in male patients with T2DM is related to SUA levels. In addition to blood glucose, more factors (e.g., uric acid and body weight) should be considered to influence sexual function.

## Figures and Tables

**Table 1:** Laboratory indices of glucose metabolism and related parameters in the study population disaggregated by quartiles of serum uric acid level.

	Group Q1	Group Q2	Group Q3	Group Q4	*p* value
*n* (%)	51 (24.9)	50 (24.4)	53 (25.8)	51 (24.9)	
Age (years)	54.59 ± 10.69	50.68 ± 11.82	51.94 ± 11.65	45.86 ± 11.07	0.002
BMI (kg/m^2^)	23.25 ± 3.04	24.56 ± 2.48	25.09 ± 3.09	27.57 ± 4.17	<0.001
Smoking (%)	27 (11.7)	23 (11.2)	16 (7.8)	23 (11.2)	
Waist (cm)	87.11	88.92	90.40	93.00	<0.001
Systolic BP (mmHg)	129.00	129.09	122.75	130.00	0.157
Diastolic BP (mmHg)	79.69	79.29	79.46	81.25	0.122
HbA_1c_ (%)	10.77	10.32	9.54	8.30	0.003
FBG (mmol/L)	8.70	8.23	8.09	7.91	0.220
PBG (mmol/L)	14.99 ± 2.83	15.31 ± 2.95	15.07 ± 3.48	15.44 ± 3.95	0.899
BUN (mmol/L)	5.41	4.92	5.39	4.92	0.100
Cr (mg/dL)	63.29 ± 10.87	66.44 ± 10.19	15.28 ± 3.49	15.22 ± 4.23	0.002
SUA (*μ*mol/L)	228.67	281.00	335.75	430.00	<0.001
HOMA-IR	1.88	2.36	3.20	3.41	<0.001

Data expressed as mean ± standard deviation or median. BMI: body mass index; BP: blood pressure; HbA_1c_: glycated hemoglobin; FBG: fasting blood-glucose; PBG: postprandial blood glucose; BUN: urea nitrogen; Cr: creatinine; SUA: serum uric acid; HOMA-IR: homeostasis model assessment-insulin resistance index.

**Table 2:** Sex hormone levels in the study population disaggregated by quartiles of serum uric acid levels.

	Group Q1	Group Q2	Group Q3	Group Q4	*p* value
*n* (%)	51 (24.9)	50 (24.4)	53 (25.8)	51 (24.9)	
TT (nmol/L)	15.07	14.84	13.98	11.64	<0.001
FT (nmol/L)	0.29	0.31	0.29	0.29	0.227
BT (nmol/L)	6.54	7.19	6.63	6.81	0.213
LH (mIU/L)	7.02	6.12	5.24	5.44	0.005
FSH (mIU/L)	8.02	5.96	5.86	4.71	0.001
E2 (pmol/L)	93.46	93.48	84.29	93.30	0.917
SHBG (nmol/L)	31.92	28.61	23.52	19.50	<0.001
DHEA (*μ*mol/L)	4.47	5.03	5.49	6.28	<0.001

Data presented as median. TT: total testosterone; BT: bioactive testosterone; FT: free testosterone; LH: luteinizing hormone; FSH: follicle-stimulating hormone; E2: estradiol; SHBG: sex hormone-binding globulin; DHEA: dehydroepiandrosterone.

**Table 3:** Comparison of different TT groups ($\bar{x}\pm s/median$).

	TT ≤ 8 nmol/L	8 nmol/L < TT ≤ 12 nmol/L	TT > 12 nmol/L	*p* value
SUA (*μ*mol/L)	330.5	344.5	284	<0.001
Waist (cm)	90.42	92.57	88.29	0.002
BMI (kg/m^2^)	26.77	25.64	24.16	<0.001
FBG (mmol/L)	9.12	8.09	8.36	0.330
PBG (mmol/L)	15.92 ± 2.55	14.87 ± 2.99	15.25 ± 3.52	0.517
HbA_1c_ (%)	10.13	8.70	10.30	0.080
HOMA-IR	2.38	3.60	2.17	<0.001

TT: total testosterone; SUA: serum uric acid; BMI: body mass index; FBG: fasting blood-glucose; PBG: postprandial blood glucose; HbA_1c_: glycated hemoglobin; HOMA-IR: homeostasis model assessment-insulin resistance index. Table 3 suggests that according to TT level, with 8 nmol/L and 12 nmol/L as the cutoff levels for the division of patients into three groups, the levels of serum uric acid, waist circumference, body mass index, and HOMA-IR in the low TT group were higher than those in the normal TT group.

**Table 4:** Correlation analysis of total testosterone and other indicators.

	*r*	*p* value
Age	0.146	0.036
Waist	−0.297	<0.001
BMI	−0.359	<0.001
HbA_1c_	0.059	0.400
HOMA-IR	−0.174	0.012
SUA	−0.317	<0.001

BMI: body mass index; HbA_1c_: glycated hemoglobin; HOMA-IR: homeostasis model assessment-insulin resistance index; SUA: serum uric acid. Table 4 shows the correlates of total testosterone levels.

**Table 5:** Multivariate regression analysis of male hypogonadism.

Variable	*B*	Beta	*T*	*p* value	95% CI	
					Lower limit	Upper limit
Constant	27.261	2.271	12.004	<0.001	22.783	31.738
BMI	−0.375	−0.100	−3.774	<0.001	−0.572	−0.177
SUA	−0.011	−0.004	−2.673	<0.001	−0.019	−0.003

BMI: body mass index; SUA: serum uric acid; HOMA-IR: homeostasis model assessment-insulin resistance index. Table 5 shows that BMI and SUA are risk factors for gonadal function.

## Abbreviations

SUA: Serum uric acid
T2DM: Type 2 diabetes mellitus
FBG: Fasting blood glucose
HbA_1c:_: Glycated hemoglobin
BUN: Urea nitrogen
Cr: Creatinine
TT: Total testosterone
LH: Luteinizing hormone
FSH: Follicle-stimulating hormone
SHBG: Sex hormone-binding globulin
DHEA: Dehydroepiandrosterone
FINS: Fasting insulin
BT: Bioactive testosterone
FT: Free testosterone
HOMA-IR: Homeostasis model assessment-insulin resistance index.
